# Effects of novel organoantimony compounds on the fungal pathogen Cryptococcus neoformans

**DOI:** 10.1099/jmm.0.002058

**Published:** 2025-09-08

**Authors:** Kaitlyn Cotton, Jacob A. Lieberman, Nikolay Gerasimchuk, Karen L. Wozniak

**Affiliations:** 1 Department of Microbiology & Molecular Genetics, Oklahoma State University, Stillwater, OK, USA; 2 Chemistry Department, Missouri State University, Springfield, MO, USA

**Keywords:** antimony-based antimicrobial, *Cryptococcus*, novel antifungal

## Abstract

**Introduction.** *Cryptococcus neoformans* is an opportunistic fungal pathogen that causes pulmonary cryptococcosis, or an acute or chronic infection in the lungs, and cryptococcal meningitis, an infection of the brain and spinal column, in immunocompromised individuals. Fungal infections are responsible for ~1.7 million deaths each year. In contrast to antibacterial drugs, the quantity of antifungal drugs capable of combating fungal infections remains low. With high toxicity and increased resistance to antifungals in recent years, the importance of finding new options for antifungal therapy is even more crucial.

**Hypothesis.** We hypothesized that a series of organoantimony compounds that previously exhibited antifungal activity could serve as effective antifungal drugs.

**Aim.** We aimed to evaluate the antifungal activity of these compounds and their mechanism of action.

**Methodology.** We first evaluated antifungal activity via MIC and minimum fungicidal concentration assay. Next, we evaluated cytotoxicity, followed by mechanistic studies via electron microscopy and RNA sequencing studies. Finally, we evaluated activity *in vivo* using a *Galleria mellonella* model.

**Results.** Results showed that several compounds were antifungal and also non-toxic. RNA sequencing identified several differentially regulated *C. neoformans* genes and pathways, including those associated with membrane transport and formation, ribosome biogenesis and gene expression. Scanning electron microscopy and transmission electron microscopy studies show altered morphology and cellular death following the treatment of *C. neoformans* with the compounds. Compounds had moderate efficacy in the *G. mellonella* infection model.

**Conclusion.** These studies show that organoantimony compounds are promising antifungal therapies, and more studies are currently underway to improve efficacy and narrow down their mechanism(s) of antifungal activity.

## Data Summary

The transcriptome datasets that support the findings of this article are available to the public in the NCBI BioSample database (ncbi.nlm.nih.gov/biosample/) under the accession number PRJNA1052015, samples SAMN38810556, SAMN38810555, SAMN38810554, SAMN38810553, SAMN38810552, SAMN38810551, SAMN38810550, SAMN38810549, SAMN38810548, SAMN38810547, SAMN38810540, SAMN38810539, SAMN38810538, SAMN38810537, SAMN38810536, SAMN38810535, and SAMN38810534. Further inquiries can be directed to the corresponding author.

## Introduction

Fungal infections are a large concern in the world today, with upwards of 1 billion cases and over 1.5 million deaths annually [1]. Of those fungal infections, 152,000 cases and 112,000 deaths annually in AIDS patients can be attributed to cryptococcal meningitis [2]. *Cryptococcus neoformans* is a fungal pathogen found around the world in soil and bird droppings, which can cause infections through the inhalation of yeast or basidospores [3]. While most individuals who encounter the fungus do not suffer any negative effects, those who are immunocompromised, such as patients with HIV/AIDS, those taking immunosuppressive medications to prevent transplant rejection or those on chemotherapy, can have symptomatic disease [4]. This opportunistic pathogen is known to cause pulmonary cryptococcosis, or an acute or chronic infection in the lungs, and cryptococcal meningitis, an infection of the brain and spinal column [5].

In contrast to antibiotics, the quantity of available antifungal drugs remains low. There are currently only four main classes of antifungal drugs approved for use in the USA: polyenes, azoles, echinocandins and pyrimidines [6,7]. Such a limited drug arsenal exists largely due to the high cytotoxicity of antifungals. Fungal pathogens are eukaryotic, making them much more like their human hosts than bacterial pathogens. As a result, the number of molecular targets for antifungals that do not also induce high host cytotoxicity is greatly reduced compared to those of bacteria [7]. Of these, only three drugs from these classes (polyenes, azoles and pyrimidines) are typically used to treat cryptococcal infection [8]. Caspofungin, one of the earliest approved echinocandins, is ineffective against *C. neoformans*, possibly due to a lipid flippase regulatory subunit, Cdc1, and calcineurin pathway regulation by Crm1 [9,10].

The first class of anticryptococcal antifungals, the polyenes (Amphotericin B), targets the cell membrane by binding to and sequestering ergosterol, a component of fungal membranes. This results in the dissolution of the cell membrane, leakage of intracellular components and ultimately cell death [6,11, 12]. This antifungal class leads to significant cytotoxicity, with the polyenes also affecting mammalian cells and causing nephrotoxicity [7]. Newer lipid formulations are less toxic [8], but there are still challenges to using this drug, due to the nature of administration by the intravenous route [8].

The azoles (fluconazole) bind to lanosterol 14-alpha demethylase in order to prevent the demethylation of lanosterol, thereby depleting ergosterol and producing toxic sterols in the cell membrane [13,14]. Azoles are fungistatic and only inhibit fungal growth rather than fully killing the pathogen [7,15]. They also exhibit cytotoxicity by attacking the mammalian cytochrome P450 [16]. Fluconazole is typically used as maintenance therapy in immunocompromised patients due to its fungistatic activity [8].

The pyrimidines (5-flucytosine) inhibit DNA replication or protein synthesis by becoming incorporated in DNA or RNA, respectively [17,18]. Pyrimidines have a broad range of antifungal activity and are often used in the treatment of *C. neoformans* [8,18, 19].

Rising resistance to antifungals in recent years, coupled with the toxicity of available antifungal drugs, makes it crucial to develop new antifungal therapies [20]. Recently, novel organoantimony (V) cyanoximates were developed that exhibited activity against several bacterial species and fungal pathogens *C. neoformans* and *Candida albicans* [21,22]. In this study, we further examine the biological activity of those compounds against *C. neoformans* and examine their potential as future antifungal drugs.

## Methods

### Fungal strain culture

*C. neoformans* strain H99 (serotype A, mating type α) (kind gift of John Perfect, Duke University) was recovered from 15% glycerol stocks stored at −80 °C and was subsequently plated on Yeast Peptone Dextrose (YPD) (BD Difco, Franklin Lakes, NJ) agar plates. For experiments, YPD broth was inoculated with the cultures and placed in a 30 °C shaking incubator for 18 h. The fungal cells were then pelleted by centrifugation and washed three times with 1× sterile PBS. The concentration of cells was quantified using a haemocytometer, with trypan blue used to exclude dead cells. The concentrations for the assay inocula were determined using the haemocytometer counts.

### Cell lines and media

McCoy murine fibroblast cells (CRL-1696), HeLa human cervical cells (CCL-2) and A549 human pulmonary epithelial cells (CRM-CCL-185) (ATCC, Manassas, VA) were grown in T75 tissue culture flasks (Corning) in a humidified tissue culture incubator at 37 °C, 5% CO_2_ in DMEM cell culture media (ThermoFisher) supplemented with 10% FBS (Thermo Fisher) and 100 U ml^−1^ penicillin/streptomycin (Thermo Fisher) and filter-sterilized through a 0.22 µm filter. Cells were passaged according to the manufacturer’s instructions. Prior to use in experiments, the cells were harvested, then pelleted by centrifugation and washed with sterile media three times and quantified using trypan blue exclusion on a haemacytometer.

### Compounds

**Fig. 1.**
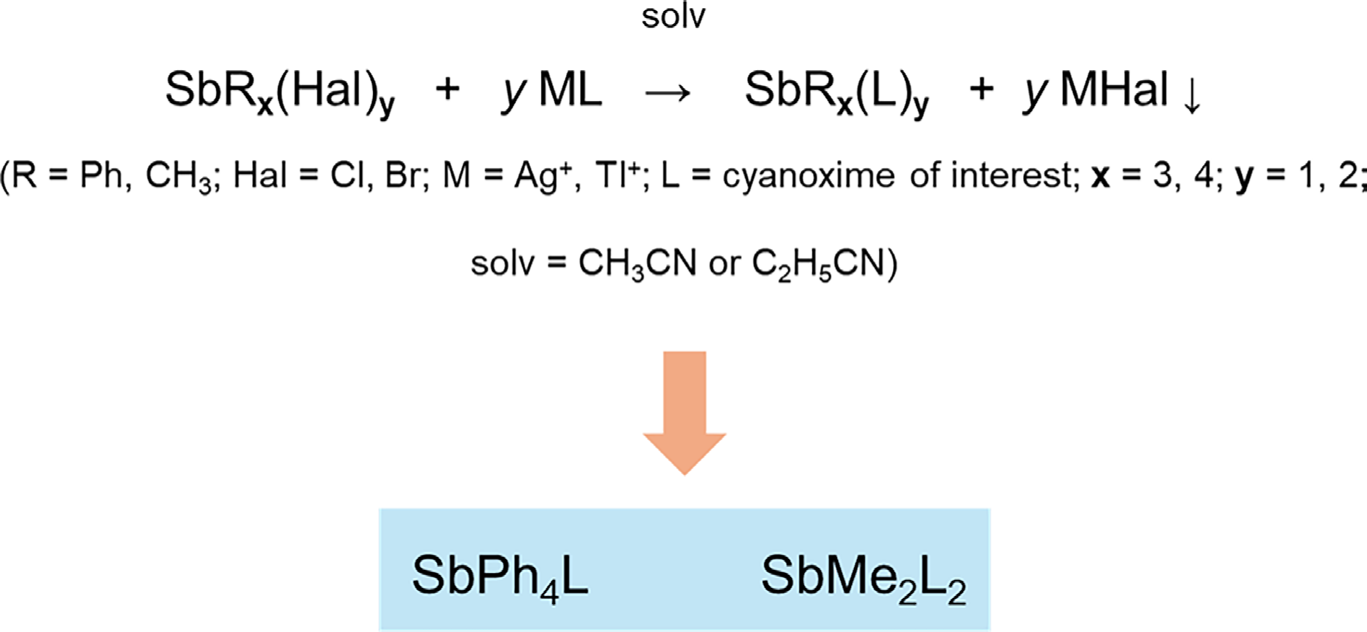
General scheme of reaction route to organoantimony(V) cyanoximates.

**Fig. 2.**
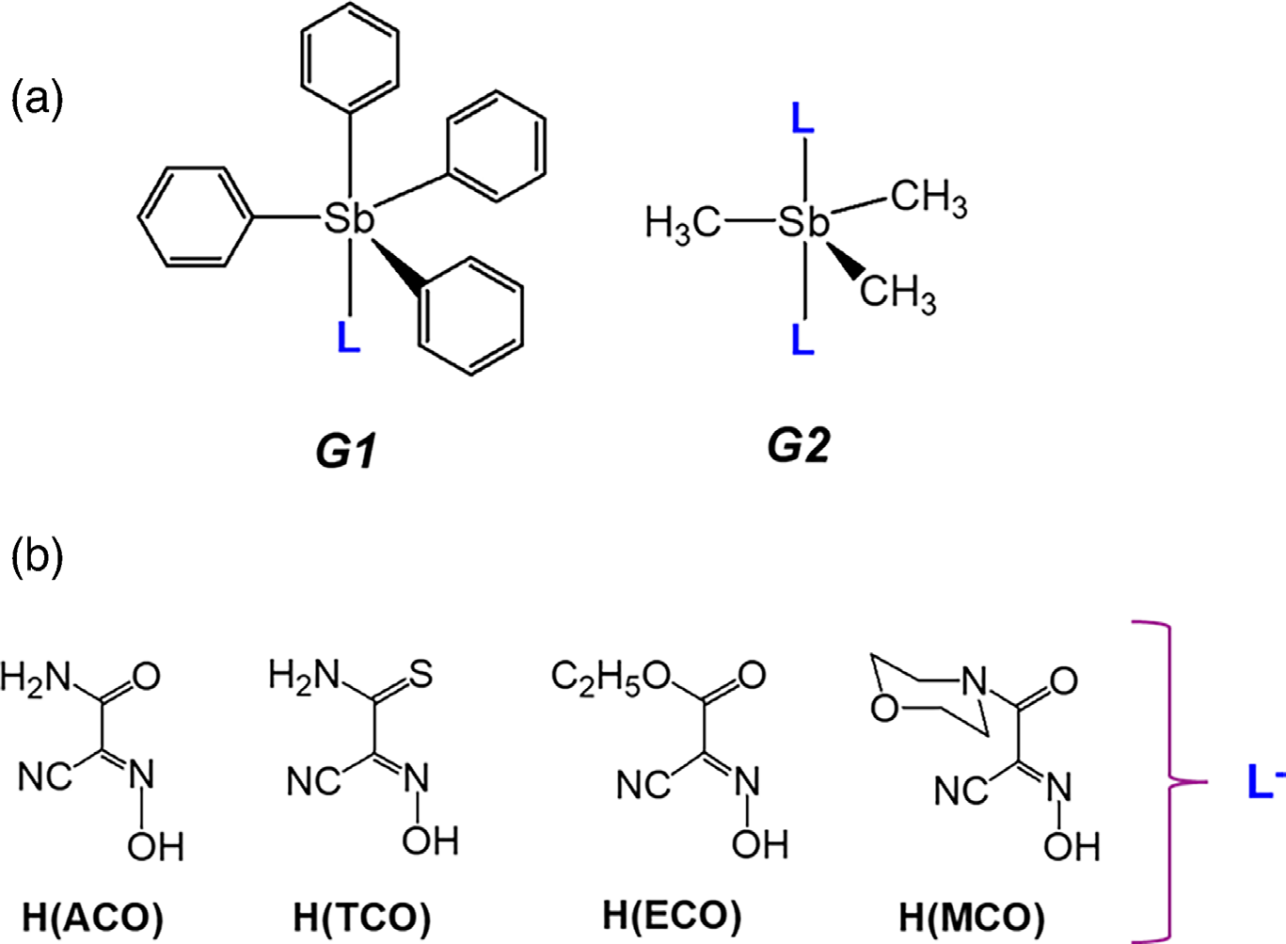
Novel antimicrobial organoantimonials showing structure graphs for ***G1*** and ***G2*** families (**a**) and particular cyanoximes used in current studies as anions l- (**b**).

**Fig. 3.**
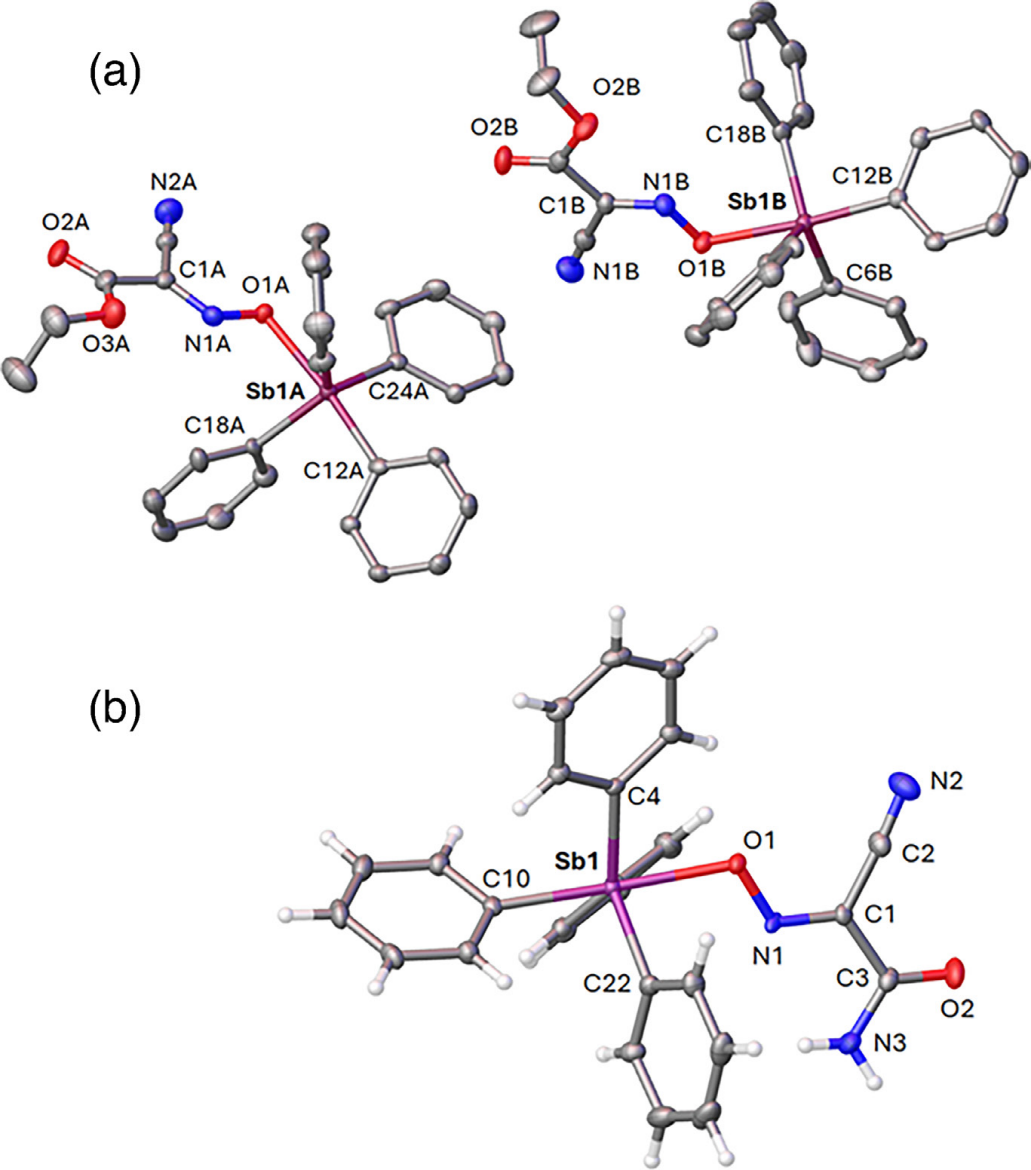
Molecular structures and numbering schemes for principal atoms for compounds of the ***G1*** family: (a) SbPh_4_(ECO), which contains two independent molecules in the ASU with H-atoms being omitted for clarity, and (b) SbPh_4_(ACO). Atomic thermal ellipsoids are shown at their 50% probability level.

Novel organoantimony compounds were synthesized by Dr. Nikolay Gerasimchuk (Missouri State University, Springfield, MO) using the heterogeneous metathesis reaction displayed in Fig. 1. This is a convenient room temperature preparation leading to high-yield preparation of a variety of organoelemental compounds of Sn(IV) [23,24], Te(IV) [25] and lately Sb(V) [21,22, 25]. The latter group of organoantimonials received considerable attention in recent years due to the discovered antimicrobial properties in two families of compounds that were given the assignments ***G1*** and ***G2*** (Fig. 2). In this work, we used four cyanoxime ligands which were found to be active in recently carried out studies [25] and lately Sb(V) [21,22, 25]. Crystal structures of all studied herein compounds are shown in Figs3  4. Novel organoantimonials represent molecular compounds with covalent bonding between a metalloid centre and phenyl and methyl groups and cyanoxime moieties. The latter demonstrate monodentate O-bonding to the Sb(V) central atoms, contrary to complexes of these anions with transition metals where cyanoximes form five-membered chelates [26,28]. Both ***G1*** and ***G2*** families of compounds are soluble in most organic solvents and form molecular solutions, thermally stable in the solid state upon heating to ~130 °C. These new compounds were tested to determine their fungistatic and fungicidal properties [21,22]. For these studies, the powdered compounds were reconstituted in cell culture-grade DMSO to 5 mg ml^−1^ and later diluted to working concentrations with either RPMI-MOPS [RPMI 1640 (Thermo Fisher) supplemented with 0.165 M MOPS (Sigma-Aldrich) and brought to a pH of 6.9–7.0, filter-sterilized through a 0.22 µm filter] or cell DMEM-based culture media (described above for cell culture of cell lines), depending on the experiment.

**Fig. 4.**
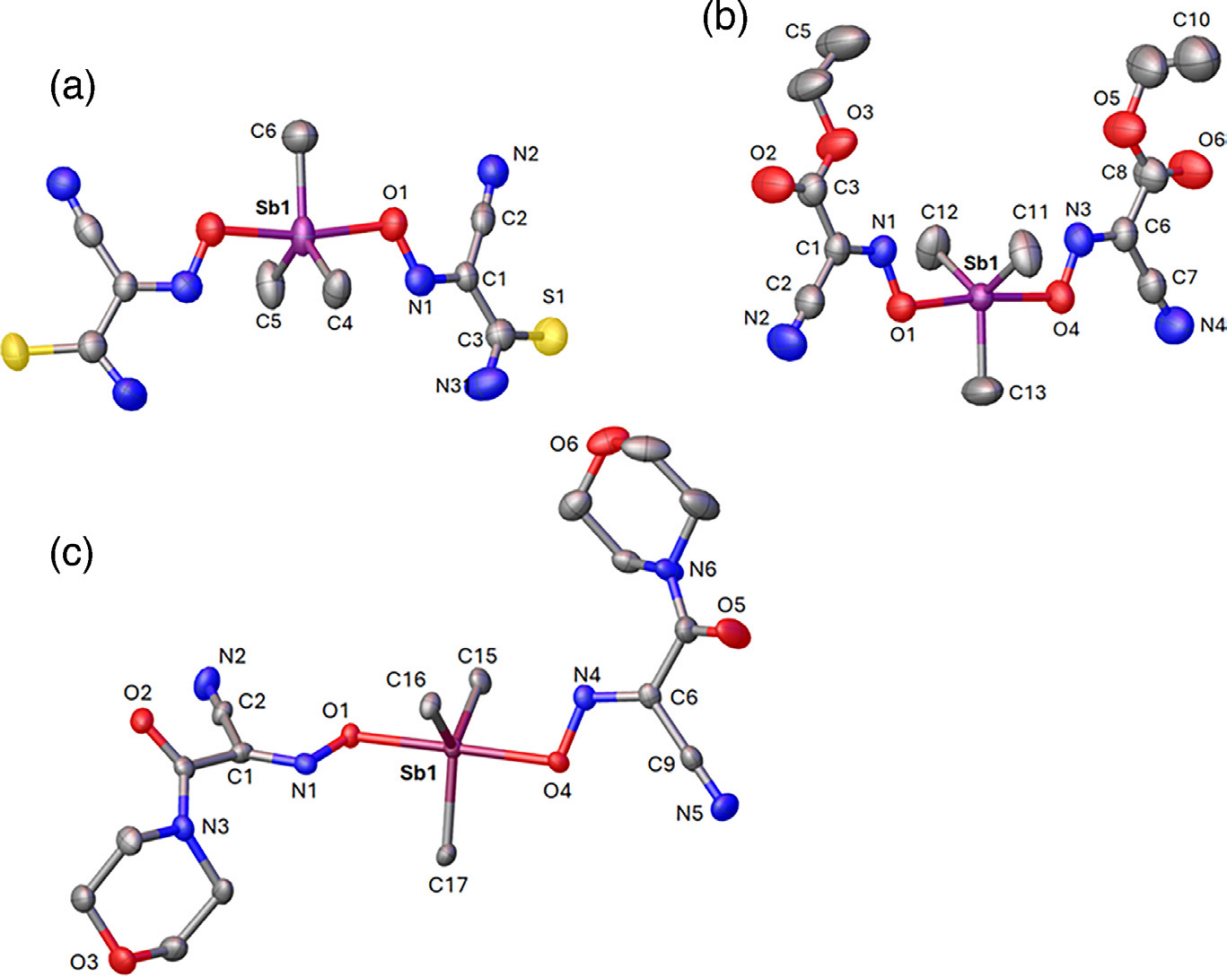
Molecular structures and numbering schemes for principal atoms in the ***G2*** family of compounds: (a) SbMe_3_(TCO)_2_, (b) SbMe_3_(ECO)_2_ and (c) SbMe_3_(MCO)_2_. H-atoms were omitted for clarity. Thermal ellipsoids are shown at their 50% probability level.

### MIC assay

MIC assays with these compounds used RPMI-MOPS media. *C. neoformans* cells were incubated with organoantimony compounds to test each compound’s inhibitory effects using the Clinical and Laboratory Standards Institute methods for antifungal drug susceptibility as previously described [29,30]. Briefly, a 96-well plate was filled with serial dilutions of the tested compound, diluted 1 : 2 with RPMI-MOPS, ranging from 100 to 0.098 μg ml^−1^. Each row on the plate served as a replicate, and controls included a negative control containing only RPMI-MOPS, a positive control for fungal growth containing RPMI-MOPS and fungal cells and a control containing only the tested compound diluted in RPMI-MOPS. A concentration of 0.5×10^3^ cells ml^−1^ of *C. neoformans* (also diluted in RPMI-MOPS) was mixed into each well of the plate. The plate was then incubated in a humidified incubator at 35 °C for 48 h. Following incubation, the wells were resuspended, and visual inspection was conducted, and an OD at 490 nm was measured on a plate reader (BioTek, Winooski, VT) to determine MIC. The concentration of compound containing no visible growth (no turbidity by visual inspection and no increased OD) was determined to be the MIC. For microscopy and RNA-sequencing experiments, which require a larger quantity of cells for either RNA extraction or visualization by electron microscopy (described below), the MIC for a higher concentration of cryptococcal cells (10×10^6^ ml^−1^) was determined by the method above.

### Minimum fungicidal concentration determination

If an MIC was found, the contents from the MIC concentration and at least two concentrations above, up to 100 µg ml^−1^ (2× MIC, 4× MIC, etc.) were plated on a YPD plate. For example, if the MIC value was 25 µg ml^−1^, then the contents from wells containing 25 µg ml^−1^, 50 µg ml^−1^, and 100 µg ml^−1^ were plated. The plates were incubated for 48 h at 30 °C. Following incubation, the number of colony-forming units (CFUs) on each plate was counted. The minimum fungicidal concentration (MFC) was the concentration of the column corresponding to the plate with no fungal growth. If all of the plates had growth, then either the compound did not have an MFC, or it was greater than 100 µg ml^−1^.

### Cytotoxicity assay

HeLa cells, McCoy cells or A549 epithelial cells at a concentration of 1.0×10^6^ cells ml^−1^ were incubated in cell culture media in culture conditions described above with each organoantimony compound at the MIC concentration, 2× MIC or 10× MIC for 24 h. Cell culture media alone and untreated cells served as negative controls, and cells treated with 100× cell-lysis buffer were used as the positive control for lysis. The Vybrant Cytotoxicity Assay (Thermo Fisher, Waltham, MA) was used according to the manufacturer’s instructions to determine per cent cytotoxicity. Per cent cytotoxicity below 30% was considered non-toxic [31].

### RNA extraction and sequencing

*C. neoformans* cells at a concentration of 10×10^6^ cells ml^−1^ were incubated with different organoantimony compounds at the 10×10^6^ cells ml^−1^ MIC concentrations (as described above) for 24 h at 35 °C in a humidified incubator, with untreated fungal cells serving as the controls. The 24 h time point was chosen to ensure that living cells remained for RNA collection. RNA was then extracted and purified using the AllPrep Fungal DNA/RNA/Protein kit (Qiagen, Hilden, Germany) using the manufacturer’s instructions for the quick-start protocol. RNA purity was verified via 260/280 OD measurements on the Take5 plate using a plate reader (BioTek). RNA was sent to Novogene (Novogene Corp, Sacramento, CA) for bulk RNA sequencing. RNA sequencing was conducted using SMARTer Stranded V2 library prep, and samples were sequenced on the Illumina Platform (PE150 Q30 ≥80%) (Novogene Corp). Gene expression was compared between treated fungal cells (treated with each antifungal compound) and untreated control fungal cells. Significant differences within gene expression were calculated using read counts adjusted by trimmed mean of M-values, and then, differential significant analysis was performed using the edgeR package (Bioconductor, Fred Hutchinson Cancer Research Center, Seattle, WA) within R Studio [Delaware Public Benefit Corporation (PBC), Boston, MA], with the significant criterion being both q value<0.005 and |log2(fold change)|>1 [32]. Genes with significantly different expression values were grouped into signalling pathways using KEGG Pathway Analysis (Kanehisa Laboratories, Kyoto, Japan) and GO enrichment analysis (Gene Ontology Consortium).

### Scanning electron microscopy

*C. neoformans* cells at a concentration of 10×10^6^ cells ml^−1^ were incubated with each antifungal organoantimony compound at the MIC concentration for 4, 8 and 12 h. The 4, 8 and 12 h time points were chosen to allow the compounds’ effects on the fungal cells to be viewed at multiple time points throughout treatment while still ensuring the survival of some fungal cells, since most fungal cells were killed by 24 h incubation. Following incubation, the samples were washed three times with a 0.2 M cacodylate-buffered wash and fixed for a minimum of 2 h in a 0.1 M cacodylate and 2% glutaraldehyde fixative. The sample was washed again, rinsed three times with the buffered wash and fixed in 10% osmium tetroxide (OsO_4_) for 1 h. Following another set of three buffered wash rinses, the samples were dehydrated with a series of ethanol washes (50%, 70%, 90%, 95% and 100%). Two hexamethyldisilane washes were performed before the samples were mounted on stubs and given an Au-Pd coat at the OSU Microscopy Laboratory. Samples were then imaged at 20,000× magnification using a FEI Quanta 600 field-emission gun Environmental Scanning Electron Microscope with a Bruker EDS X-ray microanalysis system and HKL EBSD system. At least five fields per condition per time point were examined.

### Transmission electron microscopy

*C. neoformans* cells at a concentration of 10×10^6^ cells ml^−1^ were incubated with each antifungal organoantimony compound at the MIC concentrations for 4, 8 and 12 h. Following incubation, the samples were washed three times with a 0.2 M cacodylate-buffered wash and fixed for a minimum of 2 h in a 0.1 M cacodylate and 2% glutaraldehyde fixative. The sample was washed again, rinsed three times with the buffered wash and fixed in 1% OsO_4_ for 1 h. Following another set of three buffered wash rinses, the samples were dehydrated with a series of ethanol washes (50%, 70%, 90%, 95% and 100%). The samples were washed with propylene oxide three times and then left in a 1 : 1 propylene oxide poly/bed for 12 h. Following this, cells were embedded and sliced to a thickness of 80 nm by the staff of the OSU Microscopy Laboratory. Samples were then imaged at 6,000× magnification using a JEOL JEM-2100 Scanning Transmission Electron Microscope with the Bruker Quantax 200 energy-dispersive X-ray microanalysis (EDS) system. At least five fields per condition per time point were examined.

### *Galleria mellonella* infection model

*G. mellonella* larvae were purchased from Carolina Biological Supply, Burlington, NC. After 1 day of acclimation, larvae were separated into groups of ten and injected in the last proleg with *C. neoformans* strain H99 at a concentration of 1×10^4^ cells ml^−1^ in 1× PBS, in a volume of 10 µl [33,35]. Control groups were also injected with 10 µl of 1×10^4^ ml^−1^ heat-killed H99 or 1× PBS. Following a 2 h incubation period at room temperature, the larvae were injected with 10 µl of the selected compound at the desired concentration (roughly 7.5×, 10× and 12.5× the *in vitro* MIC) diluted in PBS (treatment) or 10 µl PBS (control) in the second to last proleg [34,36]. The 10× concentration was chosen based on a study by Kay *et al*. that used 10× the *in* vitro MIC to perform a *G. mellonella* infection model, and 7.5× and 12.5× concentrations were also tested to look at a concentration both above and below 10× [35]. The larvae were incubated at 37 °C, and survival was measured for 10 days. Every 12 h, survival was checked, and cocoons were removed to arrest the *G. mellonella* in their larval stage for the duration of the experiment [37]. * G. mellonella* larvae were deemed dead following full-body melanism and immobility [35].

### Data analysis

GraphPad Prism version 5.00 for Windows was used to create graphs and conduct statistical analyses. One-way ANOVA with Tukey’s multiple comparison test (to compare pairs of columns) was used to compare data between three groups, and an unpaired t-test was used to compare data between two groups. The log-rank test was used to compare the survival of groups in the infection model.

## Results

### Several organoantimony compounds inhibit *C. neoformans* growth

To determine the antifungal activity of the organoantimony compounds, MIC assays were performed. The MIC values ranged from 2.6 µg ml^−1^ to greater than 100 µg ml^−1^, with compounds SbPh_4_(ACO), SbPh_4_(MCO), SbPh_4_(ECO), SbMe_3_(MCO)_2_, SbMe_3_(TCO)_2_ and SbMe_3_(ECO)_2_ being most effective at inhibiting *C. neoformans* growth with MIC concentrations of 18.75, 19.79, 10.94, 2.60, 12.5 and 20.83 µg ml^−1^, respectively. H(TDCO) was too unstable to be tested. All MIC/MFC experiments were conducted three to four times each (Table 1).

**Table 1.** MICs and MFCs for each of the compounds and their controls tested with *C. neoformans*

Compound	MIC (µg ml)	MFC (µg ml)	Compound	MIC (µg ml)	MFC (µg ml)
SbPh_4_(ACO)	18.75±7.22	100±0.0	Sb(Ph4)Br	41.67±14.43	>100
SbPh_4_(MCO)	19.79±16.02	100±0.0	Sb(Me_3_)Br_2_	>100	>100
SbPh_4_(TCO)	25±0.0	>100	Na[H(ACO)_2_]	>100	>100
SbPh_4_(TDCO)	33.33±14.43	>100	H(MCO)	>100	>100
SbPh_4_(ECO)	10.94±3.13	100±0.0	H(TCO)	33.33±14.43	50±0.0
SbMe_3_(2-ClPhCO)_2_	>100	>100	H(TDCO)	–	–
SbMe_3_(2,4-diClPhCO)_2_	>100	>100	H(ECO)	>100	>100
SbMe_3_(2,6-diClPhCO)_2_	100±0.0	>100	H(2-ClPhCO)	25±0.0	>100
SbMe_3_(MCO)_2_	2.60±0.9	33.33±14.43	H(2,4-diClPhCO)	25±0.0	100±0.0
SbMe_3_(TCO)_2_	12.5±0.0	50±0.0	H(2,6-diClPhCO)	50±0.0	>100
SbMe_3_(TDCO)_2_	>100	>100	-	-	-
SbMe_3_(ECO)_2_	20.83±7.22	>100	-	-	-

MIC/MFC is an average of the individual MICs/MFCs±sd.

### Two compounds are fungicidal against *C. neoformans*

To determine if the antifungal activity of these compounds is fungistatic or fungicidal, MFC assays were performed. Results indicate that SbMe_3_(MCO)_2_ and SbMe_3_(TCO)_2_ were fungicidal against *C. neoformans* with MFCs of 33.33 and 50 µg ml^−1^, respectively (Table 1).

### Compounds are non-cytotoxic to mammalian cells at the MIC concentration

**Fig. 5.**
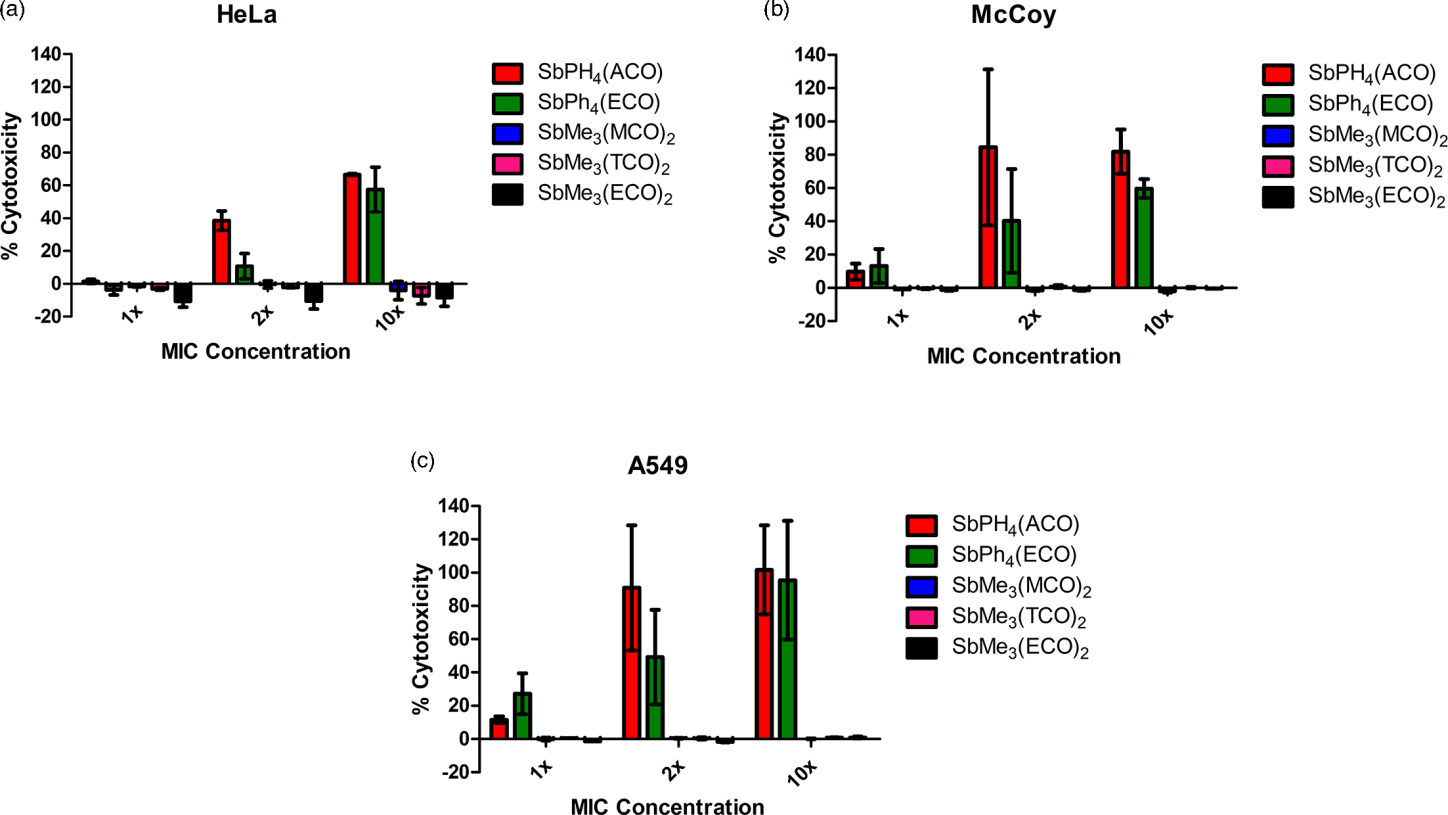
Cytotoxicity of experimental compounds. Cytotoxicity was performed using HeLa (**a**), McCoy (**b**) and A549 (**c**) cells treated with compounds at the MIC concentration, or 2× or 10× the MIC concentration. Each experiment was conducted in triplicate, and the data shown are the means±sem of the three independent experiments. All compounds exhibited low cytotoxicity (<30%).

To determine the cytotoxicity of the experimental compounds towards mammalian cells, each cell line was tested with the antifungal compounds. All compounds [SbPh_4_(ACO), SbPh_4_(ECO), SbMe_3_(MCO)_2_, SbMe_3_(TCO)_2_ and SbMe_3_(ECO)_2_] at 1× the MIC had mean cytotoxicity percentages below 30% for all cell lines tested (Fig. 5), indicating that they were non-toxic at the MIC concentration [31]. At higher MIC values, the cytotoxicity increased for SbPh_4_(ACO) and SbPh_4_(ECO) in all cell lines. The remaining compounds retained extremely low cytotoxicity in all cell lines even at 2× and 10× the MIC values, indicating that these compounds are non-toxic to mammalian cells [31].

### Treatment with compounds affects gene expression of *C. neoformans*

**Fig. 6.**
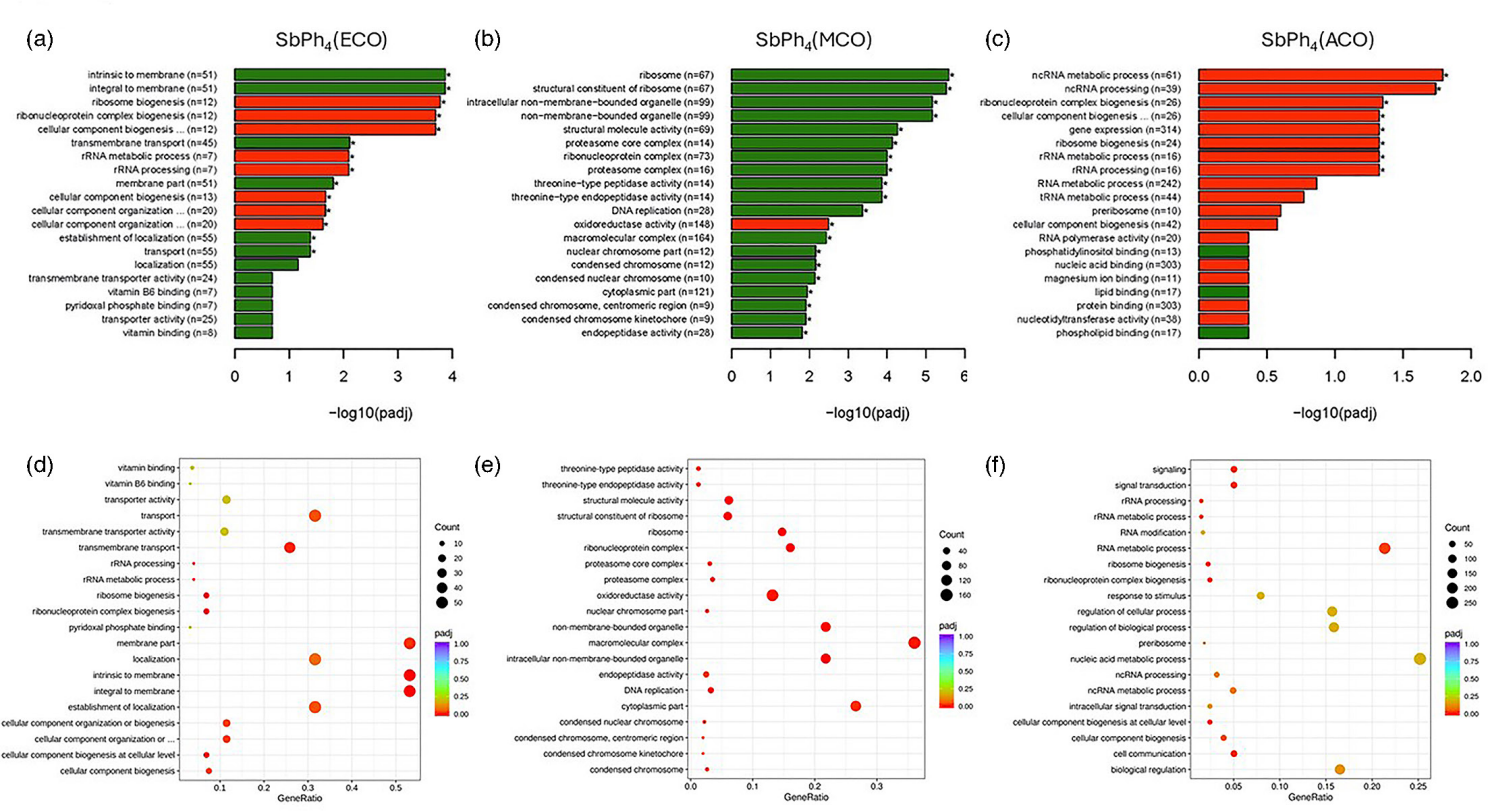
Pathways up- and down-regulated in *C. neoformans* treated with experimental compounds. RNA sequencing of untreated *C. neoformans* was compared with *C. neoformans* treated with compounds (**a, d**) SbPh_4_(ECO), (**b, e**) SbPh_4_(MCO) and (**c, f**) SbPh_4_(ACO). Pathways that were mostly up-regulated are shown in green, while pathways that were mostly down-regulated are shown in red. Data from SbPh_4_(MCO) and SbPh_4_(ACO) are compiled data from the three independent experiments. Data from SbPh_4_(ECO) represent a single experiment. * indicates a significant difference between gene expression of treated and untreated *C. neoformans* cells (*P*<0.05).

In an effort to understand how these antifungal compounds affect gene expression by the fungal organism, RNA sequencing was performed to determine the difference in the gene expression between treated and untreated *C. neoformans* cells. Novogene analyses identified 4,027 genes that were differentially expressed following treatment with SbPh_4_(ACO), with 2,029 being up-regulated and 1,998 being down-regulated; 2,768 genes that were differentially expressed following treatment with SbPh_4_(MCO), with 1,341 being up-regulated and 1,427 being down-regulated; and 742 genes that were differentially expressed following treatment with SbPh_4_(ECO), with 349 being up-regulated and 393 being down-regulated. The top 20 differentially expressed genes (by *P*-value) for SbPh_4_(ACO), SbPh_4_(MCO) and SbPh_4_(ECO) are listed in Tables2^ 3^ and 4, respectively. Using GO enrichment analysis, we further identified multiple signalling pathways that were significantly (*P*<0.05) up- or down-regulated in the treated *C. neoformans* cells (Fig. 6). Treatment with SbPh_4_(ECO) resulted in the significant (*P*<0.05) up-regulation of pathways associated with membrane transport and membrane formation, and the downregulation of pathways associated with ribosome biogenesis and rRNA processing (Fig. 6a, d). Treatment with SbPh_4_(MCO) resulted in the upregulation of pathways associated with peptidase activity, organelle and ribosome formation and DNA replication, and the downregulation of an oxidoreductase pathway (Fig. 6b, e). Finally, treatment with SbPh_4_(ACO) resulted in the downregulation of pathways associated with gene expression, RNA processing and ribosome biogenesis (Fig. 6c, f).

**Table 2.** Top 20 differentially expressed genes in *C. neoformans* treated with SbPh_4_(ECO) in order of smallest to largest *P*-value

*Gene ID*	*Log2 fold change*	*P-value*	*P-adjusted*	*Gene description*
CNAG_05305	−7.8366629	2.86E-78	2.17E-74	Aspartyl protease
CNAG_01960	3.44916049	1.26E-64	4.78E-61	Efflux protein EncT
CNAG_12403	−8.5752871	1.24E-57	3.15E-54	Aspartyl protease
CNAG_02087	2.57946416	9.54E-38	1.81E-34	Sphingosine *N*-acyltransferase
CNAG_03485	2.47279686	8.29E-30	1.26E-26	Polyamine transporter
CNAG_02347	3.39229247	5.17E-29	6.55E-26	Hypothetical protein
CNAG_00559	2.44964682	2.29E-28	2.49E-25	Hypothetical protein
CNAG_12451	3.18300673	1.03E-26	9.76E-24	ncRNA
CNAG_05279	2.89350609	1.82E-22	1.54E-19	Hypothetical protein
CNAG_06119	2.2431748	2.73E-22	2.01E-19	Spermine transporter
CNAG_00895	−3.1832894	2.91E-22	2.01E-19	Zinc transporter
CNAG_04622	1.95012279	9.85E-22	6.24E-19	Endoplasmic reticulum protein
CNAG_03713	2.64731174	3.18E-21	1.86E-18	Efflux protein EncT
CNAG_03716	−2.7381183	3.68E-21	2.00E-18	Allergen Asp f 7
CNAG_03759	2.67482313	6.55E-21	3.32E-18	Conidiation-specific protein 6
CNAG_06066	4.45360721	4.25E-20	2.02E-17	NmrA-like family
CNAG_03523	2.26944757	2.30E-19	1.03E-16	Solute carrier family 25, member 38
CNAG_00869	2.07069389	6.56E-19	2.77E-16	ABC transporter
CNAG_00876	−2.3899097	1.33E-18	5.32E-16	Ferric-chelate reductase
CNAG_12951	−2.6655965	1.80E-18	6.85E-16	ncRNA

**Table 3.** Top 20 differentially expressed genes in *C. neoformans* treated with SbPh_4_(MCO) in order of smallest to largest *P*-value

*Gene ID*	*Log2 fold change*	*P-value*	*P-adjusted*	*Gene description*
CNAG_05279	5.17482694	4.93E-94	3.69E-90	Hypothetical protein
CNAG_02548	−2.7768205	2.88E-64	1.08E-60	Cobalamin synthesis protein
CNAG_04096	−3.8340792	7.49E-59	1.87E-55	Racemase
CNAG_02959	2.20957062	5.92E-55	1.11E-51	High-affinity iron transporter
CNAG_01846	−3.3915124	4.38E-51	6.57E-48	Flavoprotein
CNAG_01953	3.54938209	3.37E-40	4.20E-37	Hypothetical protein
CNAG_01138	−2.5246072	3.82E-35	4.09E-32	Cytochrome c peroxidase, mitochondrial
CNAG_02083	2.8731227	7.76E-35	7.26E-32	Siderophore iron transporter
CNAG_03115	1.75921139	4.38E-33	3.64E-30	Hypothetical protein
CNAG_03101	1.6146825	1.89E-32	1.41E-29	Efflux protein EncT
CNAG_06761	2.04516507	2.76E-29	1.88E-26	Siderophore iron transporter
CNAG_03498	1.84886077	2.56E-25	1.60E-22	Ferric-chelate reductase
CNAG_01019	−1.3270797	3.53E-24	2.04E-21	Superoxide dismutase [Cu-Zn]
CNAG_00730	1.58701252	4.48E-23	2.40E-20	ABC transporter
CNAG_02864	−2.3209042	1.13E-22	5.64E-20	Hypothetical protein
CNAG_01954	2.22778474	9.61E-22	4.50E-19	Aldo-keto reductase
CNAG_03732	1.57288652	8.12E-20	3.58E-17	Integral membrane protein
CNAG_07309	−1.2617483	6.12E-18	2.54E-15	mRNA surveillance protein pelota
CNAG_12368	−1.6761598	9.45E-18	3.72E-15	ncRNA
CNAG_01108	−1.2760257	1.04E-17	3.72E-15	Allantoicase

**Table 4.** Top 20 differentially expressed genes in *C. neoformans* treated with SbPh_4_(ACO) in order of smallest to largest *P*-value

*Gene ID*	*Log2 fold change*	*P-value*	*P-adjusted*	*Gene description*
CNAG_01960	3.688771763	2.88E-131	2.10E-127	Efflux protein EncT
CNAG_12403	−7.915297764	2.41E-117	8.81E-114	Aspartyl protease
CNAG_03485	2.461262571	3.14E-92	7.67E-89	Polyamine transporter
CNAG_03922	3.199797714	7.53E-69	1.38E-65	NmrA-like family
CNAG_00559	2.335382234	4.46E-67	6.53E-64	Hypothetical protein
CNAG_00895	−3.185258026	8.85E-67	1.08E-63	Zinc transporter
CNAG_02548	−2.602833108	1.57E-60	1.64E-57	Cobalamin synthesis protein
CNAG_12951	−2.585526598	2.13E-54	1.95E-51	ncRNA
CNAG_05305	−8.81189906	5.26E-49	4.28E-46	Aspartyl protease
CNAG_01542	−2.101650229	2.25E-48	1.64E-45	Taurine catabolism dioxygenase
CNAG_04622	1.861039668	5.66E-46	3.76E-43	Endoplasmic reticulum protein
CNAG_02087	2.820751686	8.93E-45	5.44E-42	Sphingosine N-acyltransferase
CNAG_00919	−2.496651265	1.07E-44	6.02E-42	Serine carboxypeptidase
CNAG_03892	2.10783797	1.66E-44	8.46E-42	HSP10 (mitochondrial)
CNAG_05847	1.851548932	1.73E-44	8.46E-42	Thioredoxin-disulphide reductase
CNAG_02919	2.276425204	1.52E-42	6.96E-40	Carbon-nitrogen hydrolase
CNAG_00154	1.975787178	5.30E-42	2.28E-39	NADPH dehydrogenase
CNAG_07493	2.647003324	1.24E-40	5.04E-38	Hypothetical protein
CNAG_03716	−3.292708478	1.45E-40	5.59E-38	Allergen Asp f 7
CNAG_07492	2.794310751	2.17E-40	7.94E-38	Hypothetical protein

### Treatment with compounds results in morphological changes in *C. neoformans* cells

**Fig. 7.**
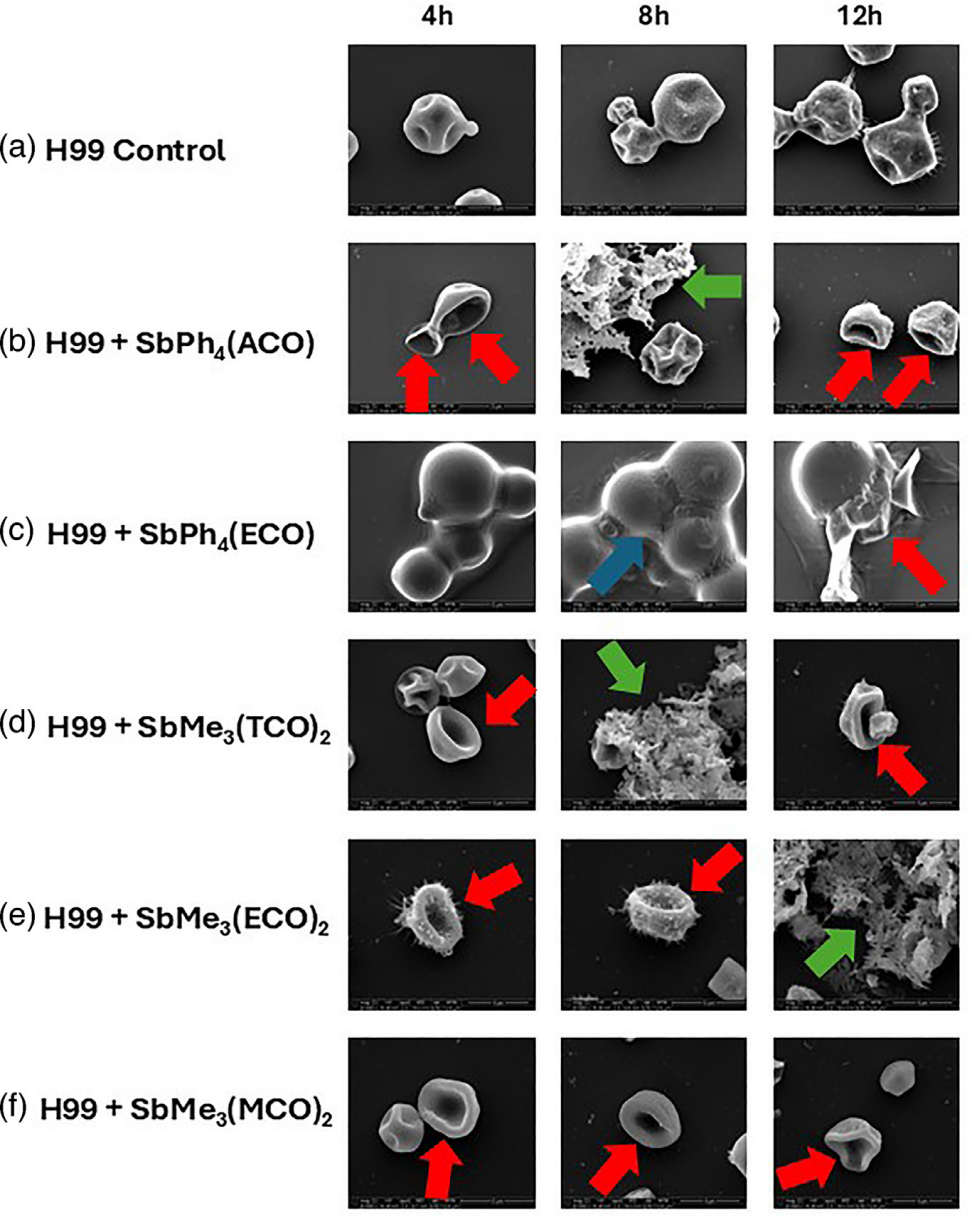
Treatment with experimental compounds results in distinct morphological changes in *C. neoformans* cells. SEM with *C. neoformans* (H99) alone (**a**), treated with SbPh_4_(ACO) (**b**), SbPh_4_(ECO) (**c**), SbMe_3_(TCO)_2_ (**d**), SbMe_3_(ECO)_2_ (**e**) or SbMe_3_(MCO)_2_ (**f**) at 4, 8 or 12 h of incubation. *C. neoformans* alone (a) exhibits a globular morphology with a distinct capsule and cell wall. Red arrows indicate cell death, green arrows indicate extracellular contents and the blue arrow indicates areas where cells did not separate after division. Images were taken at 6,000× magnification and are representative of at least 10 fields per condition and time point.

**Fig. 8.**
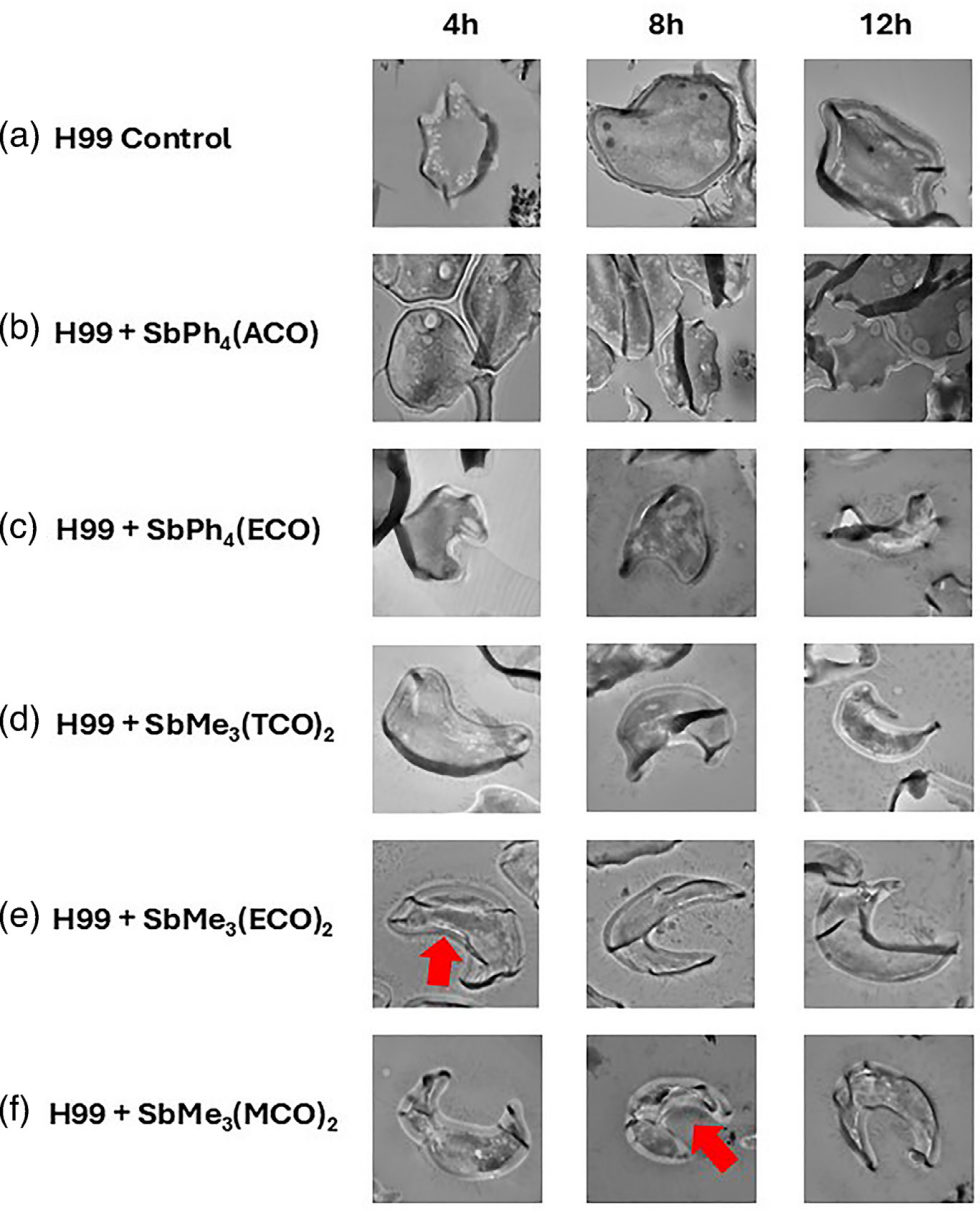
Treatment with experimental compounds results in distinct morphological changes in *C. neoformans* cells. TEM with *C. neoformans* (H99) alone (**a**), treated with SbPh_4_(ACO) (**b**), SbPh_4_(ECO) (**c**), SbMe_3_(TCO)_2_ (**d**), SbMe_3_(ECO)_2_ (**e**) or SbMe_3_(MCO)_2_ (**f**) at 4, 8 or 12 h of incubation. The red arrows point to cell death. Images were taken at 20,000× magnification and are representative of at least 10 fields per condition per time point.

In an attempt to examine the potential antifungal mechanism of action, scanning electron microscopy (SEM) (Fig. 7) and transmission electron microscopy (TEM) (Fig. 8) were performed on treated and untreated *C. neoformans* cells. The control samples (*C. neoformans* cells incubated alone) exhibit budding cells by SEM (Fig. 7a) or round cells with a fibril network radiating out from the cell capsule by TEM (Fig. 8a). SEM showed deflated-looking cells (red arrows) at multiple time points with all treatments (Fig. 7b–f), indicating cell death. Treatment with SbPh_4_(ACO) resulted in smooth capsular morphology that lacks the fibril network, as well as multiple connected cells that did not separate following cell division events (Fig. 7b). By 8 h, cracks (blue arrow) are forming in the capsule between the connected cells, and by 12 h, cell death is seen through the leakage of intracellular components into the surrounding environment (Fig. 7b). Also seen by SEM through multiple time points of samples treated with SbPh_4_(ACO) (Fig. 7a), SbPh_4_(ECO) (Fig. 7b), SbMe_3_(TCO)_2_ (Fig. 7c) and SbMe_3_(ECO)_2_ (Fig. 7d) was the presence of an unknown extracellular structure (green arrows). TEM images showed that treatment with SbPh_4_(ACO) resulted in multiple connected cells that did not separate following cell division events (Fig. 8b). In contrast, treatment with SbPh_4_(ECO) (Fig. 8c), SbMe_3_(TCO)_2_ (Fig. 8d), SbMe_3_(ECO)_2_ (Fig. 8e) and SbMe_3_(MCO)_2_ (Fig. 8f) resulted in cell death throughout all the time points, as shown by the presence of c-shaped cryptococcal cells (red arrows).

### Compounds are partially effective as antifungal therapy in a *G. mellonella* infection model

**Fig. 9.**
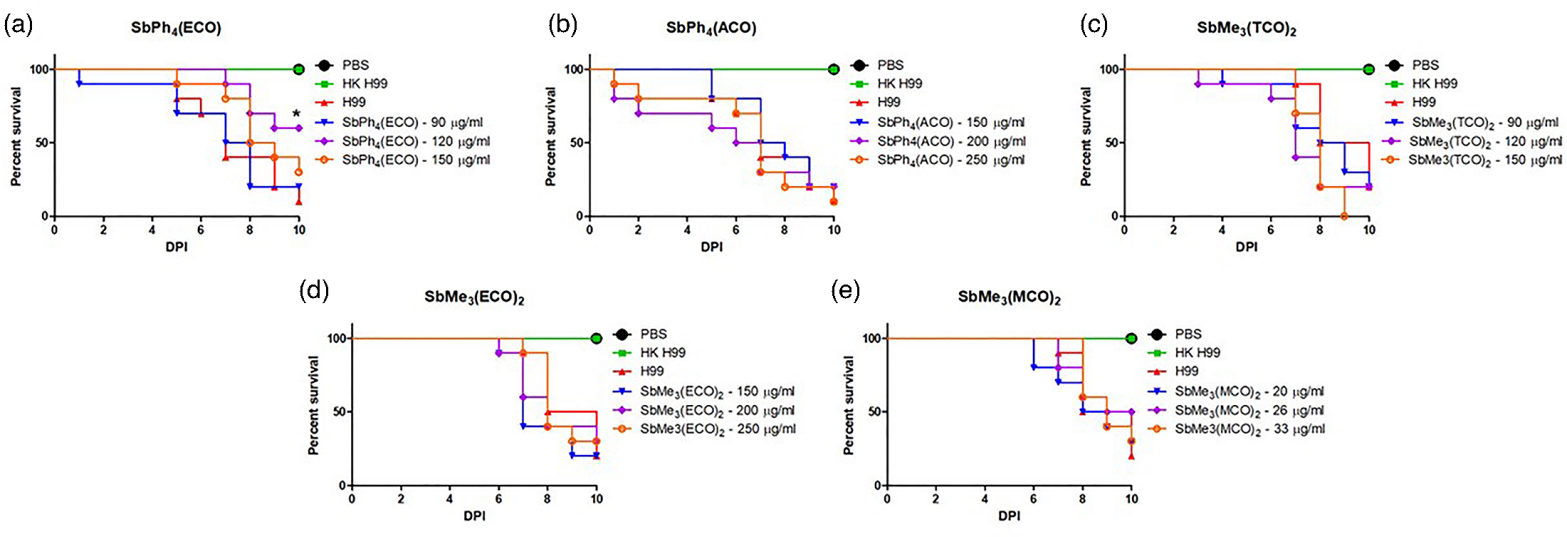
Infection model using *G. mellonella* shows *in vivo* effectiveness of only one compound against *C. neoformans* infection. *G. mellonella* larvae were treated with either 1× PBS or antifungal compounds: (**a**) SbPh_4_(ECO), (**b**) SbPh_4_(ACO), (**c**) SbMe_3_(TCO)_2_, (**d**) SbMe_3_(ECO)_2_ or (**e**) SbMe_3_(MCO)_2_. The heat-killed (non-infectious) *C. neoformans* and PBS controls resulted in 100% survival. (**a**) Treatment with SbPh_4_(ECO) at 120 µg ml^−1^ resulted in significantly longer survival than the *C. neoformans* (H99) control (*P-value<0.05). Treatment with other concentrations of SbPh_4_(ECO) (**a**), SbPh_4_(ACO) (**b**), SbMe_3_(TCO)_2_ (**c**), SbMe_3_(ECO)_2_ (**d**) and SbMe_3_(MCO)_2_ (e) resulted in similar survival as the *C. neoformans* control. Each experiment was conducted with ten *G*. *mellonella* larvae per group. * indicates a significant difference in survival between the treatment and the control groups (*P*<0.05).

To examine the effectiveness of these experimental compounds *in vivo*, a *G. mellonella* infection model with *C. neoformans* was used. Survival was monitored over 10 days to determine if any of the treatments resulted in increased survival of *G. mellonella* larvae relative to the *C. neoformans-*infected control. The negative controls (PBS-infected and heat-killed *C. neoformans*-infected larval groups) exhibited 100% survival over the 10 days. Treatment with SbPh_4_(ECO) at 120 µg ml^−1^ resulted in significantly (*P*<0.05) increased survival of *G. mellonella* larvae compared to *C. neoformans* alone (Fig. 9a). The other concentrations of SbPh_4_(ECO) tested (Fig. 9a), as well as all the concentrations of SbPh_4_(ACO) (9B), SbMe_3_(TCO)_2_ (Fig. 9c), SbMe_3_(ECO)_2_ (Fig. 9d) and SbMe_3_(MCO)_2_ (Fig. 9e) tested, were not effective at extending the life of the infected *G. mellonella* larvae relative to the control.

## Discussion

Fungal infections are a rising concern in the world today. Thanks to medical advances in the treatment of HIV/AIDS and other immunocompromising conditions, we are seeing a jump in opportunistic fungal infections in recent decades [38]. We are also seeing an increase in multi-drug-resistant fungal strains, the rise of infections by dimorphic fungal pathogens and the spread of fungal pathogens to new regions, likely due to climate change [39]. Due to the limited drug arsenal against these fungal pathogens, coupled with the high cytotoxicity of many of those existing antifungal drugs, it is important to discover new antifungal therapies [20].

Novel organoantimony(V) cyanoximates produced by Gerasimchuk *et al*. showed promising antifungal activity against * C. neoformans* and *C. albicans* [21,22]. Based on those results, we hypothesized that these organoantimony(V) cyanoximates could prove to be effective antifungals. We first confirmed that multiple organoantimony compounds [SbPh_4_(ACO), SbPh_4_(MCO), SbPh_4_(ECO), SbMe_3_(MCO)_2_, SbMe_3_(TCO)_2_ and SbMe_3_(ECO)] were effective against *C. neoformans* through an *in vitro* study, with two compounds [SbPh_4_(ACO) and SbMe_3_(MCO)_2_] exhibiting fungicidal activity.

High cytotoxicity is common in antifungal drugs due to the similarity between fungi and mammalian cells, which can be credited to their shared eukaryotic lineage and the similarity of fungal ergosterol (the target of most antifungal drugs) to mammalian cholesterol [7]. Therefore, it is important to develop antifungal drugs that are directed against molecular targets present in fungal cells, but not in mammalian cells. Our results indicate that antifungal compounds SbPh_4_(ACO), SbPh_4_(MCO), SbPh_4_(ECO), SbMe_3_(MCO)_2_, SbMe_3_(TCO)_2_ and SbMe_3_(ECO) are non-toxic to multiple cell lines (Fig. 5) and therefore likely target a cellular structure found in fungal cells, but not in mammalian cells.

Once these compounds were determined to be antifungal and non-toxic to mammalian cells, the next step was to investigate the mechanism of action behind the antifungal activity. The gene expression of untreated *C. neoformans* cells and the gene expression of *C. neoformans* cells treated with SbPh_4_(ACO), SbPh_4_(MCO) and SbPh_4_(ECO) were compared to understand the genes/pathways that were significantly up- or down-regulated following treatment. Thousands of differentially expressed genes were identified; however, our study will focus on a few genes of interest.

Following treatment with SbPh_4_(ECO), the *C. neoformans* cells exhibited downregulation of the genes CNAG_12403 and CNAG_05305, both of which encode aspartyl proteases, which have been shown to be active in acquiring nutritional requirements [40]. Also down-regulated was CNAG_02548, a gene involved in cobalamin synthesis. Cobalamin, otherwise known as vitamin B12, is involved in many metabolic pathways within the cell [41]. We saw the upregulation of CNAG_01960, CNAG_03713, CNAG_02087 and CNAG_06066. CNAG_01960 and CNAG_03713 both code for an efflux protein EncT, which is a membrane export protein. CNAG_02087 has been shown to be involved in ceramide synthesis, which forms many of the lipids found in the fungal cell membrane [42]. CNAG_06066 encodes for an NmrA-like family protein. Proteins in the NmrA family have been shown in other species to be involved with nitrogen metabolism and regulation [43]. In addition, the expression of many membrane transport proteins (including a zinc transporter, polyamine transporter and ABC transporter) was seen to be up- or down-regulated following treatment with SbPh_4_(ECO).

Treatment with SbPh_4_(MCO) resulted in the upregulation of CNAG_02959, CNAG_02083, CNAG_06761 and CNAG_03101. CNAG_02959, CNAG_02083 and CNAG_06761 all encode high-affinity iron transporters present in the cell membrane, while CNAG_03101 encodes for another efflux protein EncT. We also observed the downregulation of CNAG_02548 and CNAG_04096. As mentioned earlier, CNAG_02548 encodes a protein involved in cobalamin synthesis. CNAG_04096 encodes a racemase enzyme, which performs stereo-inversions and is involved in the formation of sphingolipids, a common membrane lipid [44]. Other up- or down-regulated genes include those involved in oxidoreductase reactions (ferric-chelate reductase, aldo-keto reductase, etc.).

Finally, treatment with SbPh_4_(ACO) down-regulated the aspartyl protease-encoding genes CNAG_12403 and CNAG_05305 and up-regulated the EncT efflux protein-encoding gene CNAG_01960, the polyamine proton transporter gene CNAG_03485 and the NmrA-like protein gene CNAG_03922, similar to SbPh_4_(ECO) [40]. This treatment also resulted in the downregulation of CNAG_02548, a cobalamin synthesis gene, as with both SbPh_4_(ECO) and SbPh_4_(MCO). Other up- or down-regulated genes include multiple enzymes involved in cellular reactions (taurine catabolism dioxygenase, NADPH dehydrogenase, carbon-nitrogen hydrolase, etc.).

Looking at the overall pathways that were up- or down-regulated following treatment with the three compounds, treatment with SbPh_4_(ECO) and SbPh_4_(ACO) resulted in the downregulation of pathways associated with ribosome biogenesis and rRNA processing, with SbPh_4_(ECO) also causing the upregulation of pathways associated with membrane transport and membrane formation. This indicates that the cryptococcal cells are diverting energy away from growth and metabolism and instead focusing that energy towards strengthening the membrane and fighting off the antifungal compound. In contrast, treatment with SbPh_4_(MCO) resulted in the upregulation of pathways associated with growth (organelle/ribosome formation and DNA replication). This is the opposite of what we saw with SbPh_4_(ECO) and SbPh_4_(ACO), and future studies will be performed to try to understand the reasoning behind this.

Overall, these RNA sequencing results indicate that these compounds may be inhibiting *C. neoformans* growth by interfering with cell metabolism, membrane production or membrane transport, decreasing the uptake of nutritional requirements while increasing the export of materials from the cell. However, more studies will need to be performed using cryptococcal mutant libraries [45,47] to narrow down exactly what genes/pathways these compounds are targeting to produce their antifungal activity.

To look into the possible mechanism(s) of antifungal activity, SEM and TEM were performed. Untreated cells appeared as globose, budding cells by SEM (Fig. 8a), all indicative of a healthy *C. neoformans* sample [48]. The electron microscopy images confirmed that organoantimony compound treatment affects the *C. neoformans* cells, and treatment with SbPh_4_(ECO) (7C), SbMe_3_(TCO)_2_ (7D), SbMe_3_(ECO)_2_ (7E) and SbMe_3_(MCO)_2_ (7F) resulted in cell death throughout all the time points, as shown by the presence of c-shaped cryptococcal cells (red arrows) [49]. We also observed the presence of an unknown structure – it is unknown whether this extracellular component contributed to the antifungal activity, or if it is just an artefact, byproduct or (most likely) compound precipitant, so more studies will be performed to determine the importance of this unknown structure. Also unknown is whether or not the cracks in the capsule of cells treated with SbPh_4_(ECO) extend through the cell wall and/or membrane of the cryptococcal cells and if they contribute to cell death.

Our findings with these organoantimony-based compounds are very novel. Current antifungal drugs under investigation, including GPI inhibitors and triterpenoids, act by inhibiting the fungal enzyme Gwt1 and 1,3*-β*-d-glucan synthesis, respectively (reviewed in [50]). Other approaches currently underway include new azoles available for inhalation, but these have only been tested with *Aspergillus* and not *Cryptococcus* infections (reviewed in [51]). But, these have the same mechanism of action as existing azoles, so this mechanism is different from our novel compounds.

Many antimicrobial treatments that are effective *in vitro* are not effective *in vivo* due to complicated interactions in living models [52]. For this reason, we wanted to test the effectiveness of the organoantimony compounds *in vivo* using a *G. mellonella* infection model with *C. neoformans*. This is an established model in the field [34]. Only SbPh_4_(ECO) at 120 µg ml^−1^ was effective at extending the survival of *G. mellonella* larvae infected with *C. neoformans*; however, whether or not this effect is biologically significant is unknown. This could be because the majority of the organoantimony compounds are not effective *in vivo*, are not effective in this particular *in vivo* model or are toxic at these higher infection model concentrations. Additional studies in the murine model of cryptococcal infection will be conducted to understand the *in vivo* potential of these compounds.

Overall, these organoantimony(V) cyanoximates, while only partially effective *in vivo* using a *G. mellonella* infection model, can provide valuable information for antifungal research. Through the identification of their mechanism of action, novel antifungal molecular targets can be identified for future research and drug production. Future studies include testing the compounds against mutant strains of *C. neoformans* that are knock-outs for the genes identified to be up- or down-regulated in RNA sequencing and cryptococcal mutant libraries [45,47] to identify genes involved in antifungal resistance and point to the antifungal mechanism, against drug-resistant strains of fungal pathogens, and in synergistic studies with existing antifungals such as Amphotericin B, since fungal infections are commonly treated with antifungals in combination.

## Abbreviations

MFC: minimum fungicidal concentration
OsO_4_: osmium tetroxide
SEM: scanning electron microscopy
TEM: transmission electron microscopy
YPD: yeast peptone dextrose
